# Improvement of PbSn Solder Reliability with Ge Microalloying-Induced Optimization of Intermetallic Compounds Growth

**DOI:** 10.3390/ma17030724

**Published:** 2024-02-02

**Authors:** Zhibo Qu, Yilong Hao, Changhao Chen, Yong Wang, Shimeng Xu, Shuyuan Shi, Pengrong Lin, Xiaochen Xie

**Affiliations:** 1 School of Integrated Circuits, Peking University, Beijing 100871, China; quzhibo1991@hotmail.com (Z.Q.); haol@pku.edu.cn (Y.H.); 2 Package R&D Center, Beijing Microelectroniscs Technology Institute, Beijing 100076, China; chenchanghao0122@163.com (C.C.); wangy@mxtronics.com (Y.W.); xsm23@mails.tsinghua.edu.cn (S.X.); 3 School of Integrated Circuits, TsingHua University, Beijing 100084, China; 4 Fert Beijing Institute, MIIT Key Laboratory of Spintronics, School of Integrated Circuit Science and Engineering, Beihang University, Beijing 100191, China; smeshis@buaa.edu.cn; 5 Department of Mechanical Engineering, Tsinghua University, Beijing 100084, China

**Keywords:** Pb-Sn solder, reliability, microalloying, intermetallic compound (IMC)

## Abstract

PbSn solders are used in semiconductor devices for aerospace or military purposes with high levels of reliability requirements. Microalloying has been widely adopted to improve the reliability for Pb-free solders, but its application in PbSn solders is scarce. In this article, the optimization of PbSn solder reliability with Ge microalloying was investigated using both experimental and calculation methods. Intermetallic compounds (IMC) growth and morphologies evolution during reliability tests were considered to be the main factors of device failure. Through first-principle calculation, the growth mechanism of interfacial Ni_3_Sn_4_ was discussed, including the formation of vacancies, the Ni-vacancies exchange diffusion and the dominant Ni diffusion along the [1 0 0] direction. The doping of Ge in the cell increased the exchange energy barrier and thus inhibited the IMC development and coarsening trend. In three reliability tests, only 0.013 wt% Ge microalloying in Pb60Sn40 was able to reduce IMC thickness by an increment of 22.6~38.7%. The proposed Ge microalloying method in traditional PbSn solder could yield a prospective candidate for highly reliable applications.

## 1. Introduction

The proven ability of solder alloys to vertically bond electronic devices and printed circuit boards with good microstructures and sufficient mechanical/electrical contact and interconnect helps to construct cross-scale interconnection in the modern electronic industry [1,2,3,4], i.e., in SMT [4,5,6], BGA [2,7] and the flip chip process [8,9,10], from board level packaging to the micro-bumps in 2.5D/3D advanced packaging [11,12]. With the development of new devices and prospect demand for products with high computational power, the package platform has continuously evolved in the past few decades, in which the requirement for solder bumps has also changed correspondingly [4,8,11]. In flip chip technology, the reduction of solder joint size and exponential increase in bump numbers has led to shrinkage of the process window and contributed to the difficulty of highly reliable welding [13]. Therefore, recent years have seen many publications concerning the process [14,15], materials [2,3], mechanism [9,16], reliability [3,17,18], testing [13,19] and other aspects of interconnect technologies.

Various solder alloys have been developed for bonding on different package levels in microelectronics [9,11], and PbSn alloy is one of the most widely used solders for its good wettability, low melting point, low cost and high reliability. In traditional solders, Pb was commonly used, but the public concern over Pb’s toxicity gradually drove the adoption of environmentally friendly lead-free soldering instead of traditional lead-containing solders [20,21]. However, the emerging new lead-free solders usually require high welding temperature, and their wettability is worse, increasing the probability of failure [15]. The higher melting temperature can harm sensitive components like plastic packages and increase the chances of tin whisker generation. Furthermore, the overgrowth of intermetallic compounds (IMCs) under high welding temperatures leads to possible brittle cracking under vibration and high-speed impact. Nowadays, PbSn solders are still used in microprocessors, servers and products for aerospace and military applications which require highly reliable interconnects against severe thermal, mechanical and electromigration pressure [15].

The high reliability of PbSn solder is dependent on the Pb content. Pb forms an eutectic microstructure with Sn, lowing the welding temperature. As a corrosion resistant component, Pb accumulates on the surface of the solder to form an oxide film, which increases corrosion resistance and the wettability of the solder. The addition of Pb improves the mechanical properties of the solder, such as elevation of impact strength and tensile strength. Pb effectively hinders the diffusion between the solder and substrate, slowing the growth rate of the IMC layer. Moreover, Pb prohibits the growth of Sn whiskers and phase transition of Sn (“Tin pest”), ensuring the normal operation of solders in a wide temperature range [4,9,15]. According to various studies, the reliability of PbSn solders increases with the Pb content, but the required welding temperature also increases synchronously [22]. Solder reactions between PbSn and the substrate were dominant to the solder wettability and IMC morphologies and thickness [23,24,25]. In reliability tests, the interconnect stability and robustness are closely related to the evolution of the IMCs’ thickness and morphologies [9]. Khatibi et al. [26] researched the evolution of interfacial IMC layer of high-lead PbSnAg/Ni and PbSnAg/Cu solder joints after aging. The Sn content of the solder and the thickness of the substrate affect the interface reliability of high-Pb solder joints. Using advanced material models, the simulated solder material revealed the IMC formation and growth procedures. With IMC accumulation at the joint interface during the reliability tests, the interconnects became more brittle when facing external shock. Bakar et al. [27] researched the IMC growth for its significant effect on solder joint reliability. They managed to increase the accuracy of the IMC layer thickness measurement to avoid the influence of hillocks and valleys. Tu et al. [9] systematically reviewed the IMC growth mechanism (morphology, thermodynamics and kinetics) of PbSn solder reactions with four metals, Cu, Ni, Au and Pd, showing the reliability decline caused by overgrowth of IMCs. In recent years, studies on the intrinsic properties of IMCs have also been continuously advancing. Cheng et al. [24] found that thin Ni_3_Sn_4_ IMC possessed a highly ductile and strain-hardening behavior despite the fact that bulk IMCs were usually considered brittle and hard. Micro/nanoscale Ni_3_Sn_4_ IMC was significantly elastically stiffer than Cu. However, the mechanical properties of the IMC layer deteriorated with the growing thickness in different reliability tests. First-principle calculations were conducted in Wang’s research [28] to reveal the physical properties of Ni_3_Sn_x_ IMC at the nickel–tin TLPS bonding layer. The calculation result showed that Ni_3_Sn_4_ IMC was the dominant risk factor among all Ni_3_Sn_x_ IMC under the stress conditions owing to its smallest deformation resistance, largest brittleness and strongest micro-cracks initiation tendency.

The drawbacks of lead-free solders in terms of higher welding temperature, lower wettability, weaker oxidation resistance and reliability, etc., have led to widespread development in various solder optimization technologies, among which the microalloying method has achieved great success for its easy implementation and good optimization effect [29,30,31,32]. Many elements have been used for microalloying, including Cu, Zn, Ni, Bi, P, Ge, Co, In, Cr and some rare-earth element like Re, and some novel solders with microalloying have been applied in industry [31]. As a commonly used microalloying element in SnAgCu (SAC) and SnCu solder alloy, Ge functions as an anti-oxidant and improves the wetting performance of the alloy. The addition of Ge reduces the aging degradation and prohibits IMC overgrowth [33,34]. Hasnine et al. [33] researched the effect of high-temperature storage on the microstructural and mechanical behavior of SnCu solder alloys with doped Ge. The addition of Ge restricted the Cu diffusion into the alloy and inhibited the IMC growth and growth mode transition, extending the package lifespan. Liu et al. [35] found that Ge microalloying improved the oxidation resistance. On the oxidized surface, compact GeO_2_ formed in the outer layer, stabilizing the surface structure and reducing the potential formation of inclusion. Zhang et al. [36] investigated the influences of Ge on SnCu solder alloy, including the optimization of tensile properties, refinement of microstructures, solid solution strengthening, oxidation protection and IMC growth inhibition. However, research on Ge microalloying in PbSn solder is sparse, despite its importance. Considering that the traditional PbSn solder still has a certain application range, especially for aerospace and military applications, it is imperative to conduct microalloying research on PbSn alloys to further improve their reliability.

In this study, the effect of Ge microalloying on the PbSn solder was studied via the comparison of solders with/without Ge content. After systematically characterizing the reliability of PbSn alloys with different compositions, the main interfacial IMC component, Ni_3_Sn_4_, was analyzed with first-principle calculation, showing its dominant formation mechanism: Ni-vacancy diffusion along the [1 0 0] direction in the Ni_3_Sn_4_ cell. Ge microalloying lifted the exchange energy barrier of Ni atoms and the vacancies, and the Ni_3_Sn_4_ growth was thus hindered. In reliability tests, the comparison of Pb59.97Sn40Ge0.03 solder and Pb60Sn40 solder implied that Ge microalloying effectively reduced the IMC thickness and inhibited the IMC growth mode transition from a planar morphology to scallop-shaped and bamboo-shoot-shaped fronts, increasing the solder reliability and lifespan. We hope the research and discoveries presented here can promote the further application of microalloying in PbSn solder alloys.

## 2. Experimental Section

### 2.1. The Fabrication of Solder Alloys

Pb-Sn alloy solders of four different compositions (90Pb10Sn, 80Pb20Sn, 60Pb40Sn, 37Pb63Sn) were fabricated via alloy melting processes illustrated in Figure S1a, in which an additional KCl-LiCl salt mixture with a weight ratio of 1.3:1 was used to protect the stoichiometrical alloys from oxidation. Before the processing, dilute hydrochloric acid was used to remove the oxide film on the surface of the Pb and Sn bulk metal. During the 2 h melting process under 600 °C in a muffle furnace (SX5-12, Shanghai Shuli Co., Ltd., Shanghai, China), the contents of the crucible needed to be stirred evenly every 15–20 min. Then, the crucible was taken out and cooled to room temperature. The solder was finally obtained after surface washing.

### 2.2. The Processing of Ni-Cu Substrate

The substrate used for wettability measurements was fabricated by electroplating Ni layer on a copper plate. The composition of the solution included 0.29 g mL^−1^ NiSO_4_, 0.045 g mL^−1^ NiCl_2_, 0.035 mL H_3_BO_3_ and 7 × 10^−5^ g mL^−1^ sodium dodecyl sulfate. After the solutes were mixed and dissolved, H_2_SO_4_ was gradually added to adjust its pH to 3. The copper plate was immersed in the solution to a depth of 6 cm. The electroplating process took 1 h with a current of 100 mA to form an even and compact Ni coating. Then, the substrate was cleaned with DI water and cut into 6 cm × 3 cm pieces.

### 2.3. Characterization of Physical Properties of the Solders

The metallographic analysis on the as-prepared solders follows a common process (mounting, grinding, polishing and observation). An optical microscope and a scanning electron microscope (SEM, Phenom Scientific, Eindhoven, The Netherlands) were used to obtain the images of the microstructures, respectively. An energy dispersive spectrometer (EDS) was employed for elemental analysis during SEM observation.

Qualitative phase analysis of the solders was performed via the X-ray diffraction method (PANalytical X’Pert Prompd) to show the phase evolution. A 1 × 1 × 0.5 cm metal block was machined from the solder alloy casting as a standard sample for the XRD test.

Tensile testing (Instron 5900 Series universal tensile machine) with a loading rate of 0.01 mm/s was conducted for quantitative analysis of the mechanical properties of the samples. The experiment used planar tensile samples whose parameters are shown in Figure S2.

Generally, the peak temperature during reflow process is 30–40 °C above the melting point. Thus, the melting points were measured with a differential scanning calorimeter (DSC, Mettler Toledo, Greifensee, Switzerland) with a heating rate of 5 °C/min, and an Al_2_O_3_ crucible was treated as reference. The DSC measurements were protected with a nitrogen flow of 50 μL/min. The sweeping gas was also nitrogen, and the flux was 20 μL/min.

**Figure 1:**
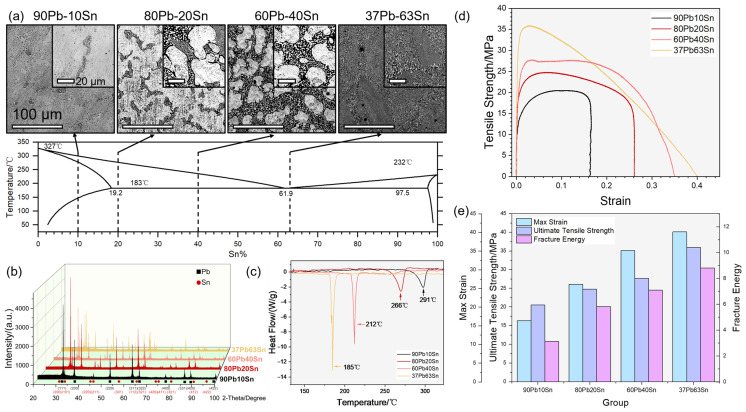
The synthesis of four different solder alloys and their performances. (**a**) The digital metallographic photos of the samples. (**b**) The variation of the XRD pattern of the composition. (**c**) The DSC patterns showing their melting point. (**d**) The stress–strain curve using standard tensile samples. (**e**) The mechanical properties derived from (**d**). The scale bars are 20 μm and 100 μm, respectively.

The wettability test was conducted using the wetting-spreading method, as illustrated in Figure 1a. The contact angles of 200 mg solder alloys on the Ni-Cu substrate were measured on an optical surface analyzer (Sindin SDC-200S); the alloy samples placed on the substate were melted in the ovens set with 10 °C temperature gradients. Each value was measured three times to get the average value in the graphs.

### 2.4. Reliability Tests

The reliability tests adopted 25D silicon interposer and low temperature co-fired ceramic substrate (LTCC) as a non-airtight package platform. The package contained more than 10,000 solder bumps, and each bump was 100 μm in size. The structure of under-bump metallization (UBM) is Ti/Cu/Ni, while the metal coatings on the ceramic substrate’s pads consisted of Ni/Au.

Generally, Pb-Sn solders are widely used in aerospace and defense products for their higher reliability than conventional lead-less solders in harsh environments facing complex physical and chemical conditions. The mostly commonly used reliability tests applied in microelectronics includes the standard temperature circulation test (−65 °C–150 °C), multiple reflow test (215 + 5 °C) and high-temperature storage test (125 °C), which were considered in this research. The details of the reliability tests can be found in the Supplementary Materials.

The mechanical strength of the flip chip package was measured using a shear force tester (DAGE4000) with a loading rate of 254 μm/s, shown in Figure S1b. After the chip was detached from the substrate, the images of the fracture morphology were obtained via 3D-field microscopy (Keyence, Osaka, Japan).

## 3. Results and Discussion

### 3.1. PbSn Solder Properties Experimental Analysis

In this section, Pb-Sn solders with different chemical compositions were systematically studied. The four different solder alloys were fabricated as depicted in Figure S1a. Protection with molten salts helps to avoid severe oxidation, maintaining the ratio of two elements in the melt. As shown in Figure 1a, the microstructures changed with the chemical compositions of the solder alloys. According to the eutectic phase diagram of Pb and Sn, both elements exist in solid solution (α-Pb phase or β-Sn phase) or eutectic (Pb:Sn = 38.1:61.9). Larger and inset images showed millimeter- and micrometer-scale perspectives, respectively. As the Sn content rose from 10% to 63%, the dark eutectic of the white α-Pb phase gradually took place in the Pb-Sn cast condition microstructures. Due to the proximity of the Pb37Sn63 solder to the eutectic, the microstructure was completely occupied by random-oriented lamellar eutectic. Correspondingly, the XRD patterns in Figure 1b show the decrease in α-Pb phase signals (black square) and the increase in β-Sn phase signals (red circle). In Figure 1c, significant changes of heat flow mark the violent melting of the alloys. Due to the inclusion of various microstructures with different melting points in alloys, they have a melting range rather than a melting point, where solid and liquid coexist. The measured “melting point” of the four samples were 291 °C, 266 °C, 212 °C and 185 °C (from 90Pb10Sn to 37Pb63Sn), respectively, each corresponding to a melting range. To access their mechanical properties, the samples underwent tensile tests, depicted in Figure 1d,e. Elastic deformation, uniform plastic deformation, local concentrated plastic deformation after necking and final fracture were observed during the tests. Among the four as-cast Pb-Sn alloys, 37Pb63Sn alloy showed the best performance in the tensile test for its maximum deformability and absorbed energy before final fracture. The excellent mechanical properties of the alloy were largely attributed to its eutectic microstructure, which showed small interlayer spacing and fine grains. Furthermore, the uniform synchronous deformation of the refined grains made the eutectic solder alloy less prone to stress concentration, delaying the initiation and propagation of cracks. Therefore, the 37Pb63Sn solder had both good plasticity and tensile strength. As the Sn content decreased, the max strain, ultimate tensile strength and fracture energy synchronously declined. Moreover, the fracture mode changed from ductile fracture for 37Pb63Sn to brittle fracture for 90Pb10Sn at the end stage of their stress–strain curves. Due to the lower melting point and obvious performance advantages under room temperature, the 37Pb63Sn was considered the mostly commonly used solder alloy containing Pb.

**Figure 2:**
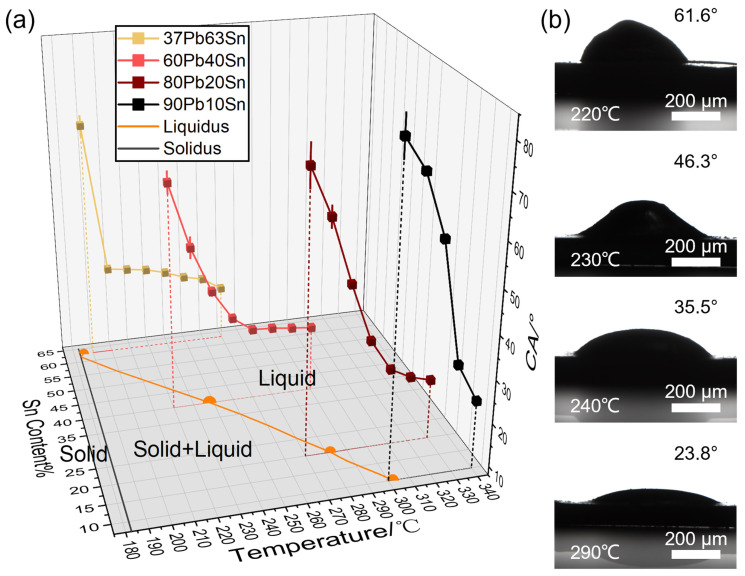
The statistics of solder wettability. (**a**) The contact angles of four solder alloys on the Ni substrate. (**b**) The images of 200 mg Pb60Sn40 solder on the Ni substrate under different temperatures.

In Figure 2, the high-temperature wettability to the Ni substrate of the four solders is systematically shown. The alloys melted in the temperature ranges between the liquidus and solidus, and their viscosity decreased as the rate of liquid phase increase during the heating process. When the alloys started to wet the substrate and showed contact angles, they were not fully melted (under the liquidus). In Figure 2a, the contact angles decreased with increasing temperature for each alloy, but the lowest contact angle was above 20°. When the temperature was close to their melting point, the receding rate of contact angle was much higher. During synchronous heating of the four solders, 37Pb63Sn first wetted the Ni substrate and then spread, ensuring a good mechanical and electrical contact, while the solders with high-Pb content required much higher ambient temperature for good welding, which consumed more energy and triggered potential failure in applications. The solders wetted the Ni substrate well at 20–40 °C above their melting point regardless of their compositions, proving the excellent welding performance of Pb-Sn alloy. In Figure 2b, the elevating temperature facilitated the spreading of Pb60Sn40 solder on the Ni substrate. Before the measurement, the polishing of the Ni substrate, purification of solder alloys and inert gas protection was necessary to prevent interfacial metal oxides from interrupting the wettability measurement.

**Figure 3:**
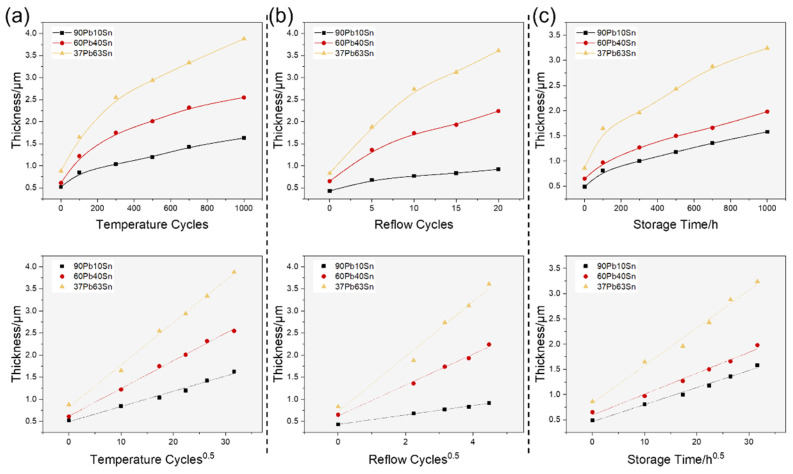
The statistics of IMC evolution during reliability tests: (**a**) the temperature circulation tests, (**b**) the multiple reflow tests and (**c**) the high-temperature storage time.

In the electronics industry, high-Pb solder has shown its higher reliability over 37Pb63Sn solder, making it commonly used in products for aerospace and military purposes. In the identically applied reliability tests, the superb stability of high-Pb solder was fully revealed. The temperature circulation test examined the ability of the device to withstand extreme high and low temperatures, as well as the impact of alternating temperature changes. The high-temperature storage test verified the reliability and quality of devices that can withstand test conditions throughout their entire lifespan, highlighting defective products with initial failure. The multiple reflow test determined whether the device could withstand the thermal effects generated during the welding process and evaluated the impact of reflow effects. The cross-section SEM images of the solder bumps are shown in Figures S3–S5 sequentially. The inset images show the full view of the solder bumps, while the large image shows the interfaces between the solder balls and the substrates. During the reliability tests, the IMC growth and development was crucial to the mechanical strength. The IMC thickness was measured from the SEM images and is organized into diagrams in Figure 3. The high-temperature procedure in the reliability tests greatly accelerated atomic diffusion, enabling the rapid formation of IMCs. Obviously, as the experiment progressed, the IMCs gradually thickened, and the growth rate declined, showing linear positive correlation The IMC thickness was linearly correlated with the square root of temperature cycles, reflow cycles and storage time, respectively. Considering that the atomic diffusion rate was also proportional to the square root of time (t^1/2^), the determining factor of IMC formation was bulk diffusion for all solders. The thickness and growth rate of IMCs was lower in solder bumps with higher Pb content, indicating that Pb effectively inhibited the growth of IMCs. Additionally, Pb also greatly affected the IMC morphology evolution. For high-Pb content solders like 90Pb10Sn, the initial planar IMCs was about only 0.5 μm in thickness and the IMCs grew slowly in the long-period reliability tests. After 1000 temperature cycles, 20 reflows or 1000 h high-temperature storage, the IMCs’ morphology slightly roughened, showing a clear trend of scalloping. Pb60Sn40 was a typical medium-temperature solder with moderate Pb content. The IMCs of the as-prepared Pb60Sn40 bumps were scallop-shaped and further developed into bamboo-shoot-shaped columns and crystals, especially in multiple reflow tests. When the Pb content was lower, such as in Pb37Sn63 solder, the bamboo-shoot-shaped IMCs grew quickly and intruded into solders with less difficulty, forming a more complex and disordered interface. The interfacial strength was low, leading to the easy dewetting and detachment of solders and final failure of the package. This phenomenon was particularly evident in reliability tests with strong convection like the multiple reflow tests. In Figure S3, clear interfacial crack across the IMCs can be observed in 37Pb63Sn solder bumps after 1000 temperature cycles. In the high-temperature storage experiments, IMCs tended to grow in layers with a much weaker tendency to form rougher interfaces. In summary, the IMC thickness increased and the IMC morphology became uneven with increasing Sn content. Considering the thickness and morphology of the IMCs, flip chip devices with 90Pb10Sn bumps have the best reliability under the same reliability test conditions.

**Figure 4:**
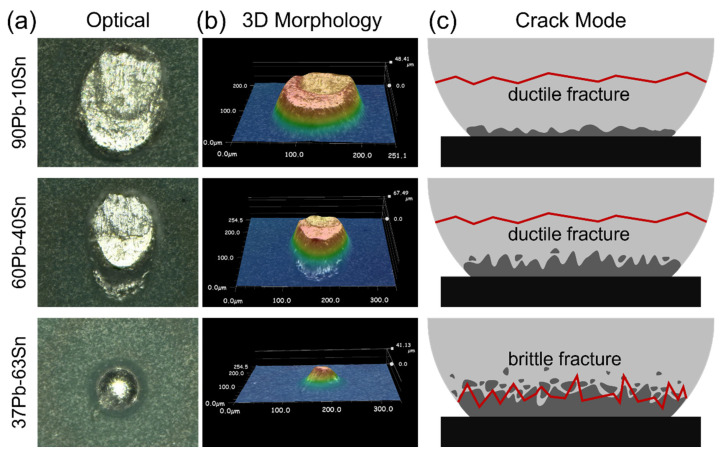
The relationship between IMC morphologies and the crack behavior under shear stress of different solders after 15 reflow cycles. (**a**) The optical and (**b**) 3D optical observation of the bumps after detachment. (**c**) The corresponding fracture mode of the three bumps.

The mechanical properties of the solder bumps were greatly related to the interfacial microstructures. Shear force applied on the flip chip detached the mounted chip from the substrate, leaving residual solder alloys. Observation of the residues shown in Figure 4a,b suggested that the 37Pb63Sn solder joints broke at the solder–substrate interface while the other two broke inside the solder ball, showing a crack mode transition from ductile fracture to brittle fracture with decreasing Pb content after identical reliability tests. Due to the prosperous growth of bamboo-shoot-like IMC microstructures, the solder–substrate interface bonding was severely cut and weakened by brittle IMCs.

**Figure 5:**
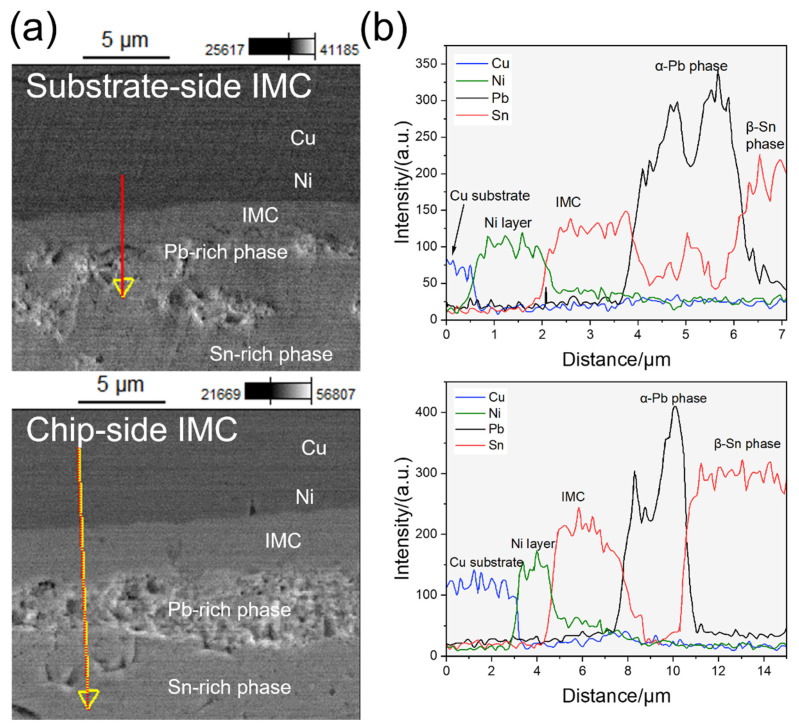
The interface structure observation. (**a**) The SEM images of the IMCs on both sides of the as-prepared 60Pb40Sn bump. (**b**) The chemical composition along the lines in (**a**) characterized with EDS.

Further observation of the solder–substrate interface revealed its microstructures and chemical compositions, as shown in Figure 5. On the electroplated millimeter-scale Ni layer, the solders wetted well and formed IMCs composed of Sn and Ni. The atomic concentration of Ni decreased in the solder. The main component of IMCs was Ni_3_Sn_4_ according to the atomic ratio of Ni and Sn, while other components, including Ni_3_Sn_2_ and Ni_3_Sn, existed close to the Ni layer, owing to the diffusion limitation. The chemical composition in the solders were not uniform due to the α-Pb phase and β-Sn phase with different element ratios.

### 3.2. Reliability Optimization Based on Microalloying

In the following section, starting from the first-principles calculation of the Ni_3_Sn_4_ growth process and doping effects of other elements, the long-term reliability of flip chip was evaluated with both experimental and simulation methods. All energy calculations were based on the Cambridge sequential total energy package (CASTEP, GGA-PBE functional) module and the valence band electrons of Sn (5s^2^5p^2^), Ni (3d^8^4s^2^) and Cu (3d^10^4s^1^), Ge (4s^2^4p^2^) were calculated using the Vanderbilt ultrasoft pseudopotential method.

**Figure 6:**
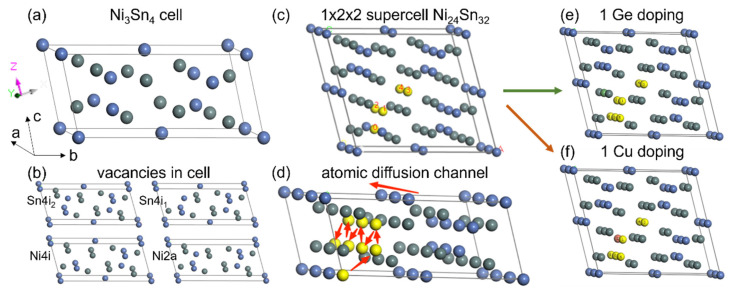
The first-principles calculation of the Ni, Cu and Ge diffusion in Ni_3_Sn_4_. (**a**) The Ni_3_Sn_4_ cell. (**b**) Different vacancies in the cell. (**c**) Establishment of 1 × 2 × 2 supercell Ni_24_Sn_32_ to calculate the diffusion behavior of Ni in the cell. (**d**) The Ni diffusion channel in the 1 × 3 × 1 supercell. Atomic doping of Ge (**e**) and Cu (**f**). The blue, grey-green, green and reddish-brown atoms are Ni, Sn, Ge and Cu atoms. The bright yellow atoms are marked Ni atoms.

The Ni_3_Sn_4_ cell is shown in Figure 6a, in which six Ni atoms and eight Sn atoms constituted this monoclinic cell, belonging to the C2/m space group. Lattice constants included a = 11.952 Å, b = 3.991 Å, c = 5.131 Å and β = 104.84°. There were two types of Ni and two types of Sn atoms in the cell, occupying positions marked as 2a and 4i and 4i_1_ and 4i_2_, respectively. The atomic coordinates are organized in Table 1.

For Ni_3_Sn_4_-based IMCs, the diffusion process was mainly related to bulk diffusion, as discussed above. The vacancy diffusion mechanism was considered dominant because the atomic radii of Sn, Pb, Ni and Cu in the transition element region were close (as shown in Table S2), making interstitial diffusion difficult for them. The exchange mechanism was sparsely observed owing to its high activation energy and thus was not considered in this study.

The Ni_3_Sn_4_ cell with four kinds of vacancies was considered as depicted in Figure 6b: Sn(4i_1_), Sn (4i_2_), Ni(2a) and Ni (4i). In this part (vacancies calculation using Geometry Optimization module), the parameters are shown in Table S1. The difference between the complete cell with no vacancy and the cell with vacancies was the formation energy of vacancies, calculation results of which are shown in Table 2, indicating that the formation of Ni vacancies was much easier than those of Sn. Additionally, the atomic radius of Ni (1.24 Å) was much smaller than that of Sn (1.58 Å), and the diffusion distance for Ni was also smaller than that of Sn in the cell. Therefore, Ni was considered the main diffusion element (Ni into PbSn solder but not Sn into Ni substrate).

To further determine the main diffusion channels in Ni_3_Sn_4_ structures, the TS Search module was applied to calculate the transition state of Ni atoms among these vacancies in the supercell constructed in Figure 6c to facilitate the linear/quadratic synchronous transit (LST/QST) calculation. All the parameters are listed in Table S3, and the exchange energy barriers between Ni atoms and Ni vacancies are all listed in the Table 3.

Obviously, the 1~0 diffusion was much easier than other diffusion types. As shown in Figure 6d, for Ni atoms in 4i positions, they diffused along a zigzag pathway along the [1 0 0] direction, while Ni atoms in 2a positions first migrated to 4i positions via other diffusion paths and then diffused like the Ni atoms in 4i positions. In summary, the main diffusion channels in Ni_3_Sn_4_ were along the [1 0 0] direction according to the calculations above.

The doping of other elements causes lattice deformation, which may interfere with the Ni diffusion procedure. The cell formation energy after Ge/Cu substitution at the four positions in the 1 × 2 × 2 supercell was considered in Table 4. The lowest cell formation energy of Ge and Cu were Ge-Sn (4i_2_) substitution (−0.25943 eV) and Cu-Ni (4i) substitution (−0.26015 eV), respectively, but the cells after doping were all less stable than the pristine Ni_3_Sn_4_ cell, indicating that the lattice deformation caused by doping elevated the lattice energy.

As the Ni atoms mainly migrate via the 1~0 diffusion along the zigzag pathway along the [1 0 0] direction in the Ni_3_Sn_4_ IMCs, the doping elements which inhibit 1~0 Ni diffusion will further lower the growth rate of the IMCs, preventing the solder bumps from failures caused by IMC overgrowth. The [1 0 0] diffusion channel is dependent on the 1~0 diffusion path, and the exchange energy barrier of the path is directly related to the cell structure, which underwent deformation after doping. In Figure 6e,f, the doping of single Ge or Cu atoms in the 1 × 2 × 2 supercell is illustrated. The calculation results of the Ni-vacancies exchange energy barrier after doping are organized in Table 5 and Table 6. After the single atom doping in the supercell, the concentrations of Ge and Cu were 1.4 wt% and 1.22 wt%, respectively.

From the calculation above, the 1~0 diffusion was still much easier than other possible paths, suggesting than the 1~0 diffusion was dominant after doping. Comparison of the minimum exchange energy barrier with Ge/Cu doping and the pristine Ni_3_Sn_4_ cell showed that the Ge substitution increased the required energy for Ni-vacancies exchange while the Cu substitution facilitated the Ni-vacancies exchange; in other words, Ge was a potential IMC growth inhibiter while Cu doping accelerated the IMC growth rate.

**Figure 7:**
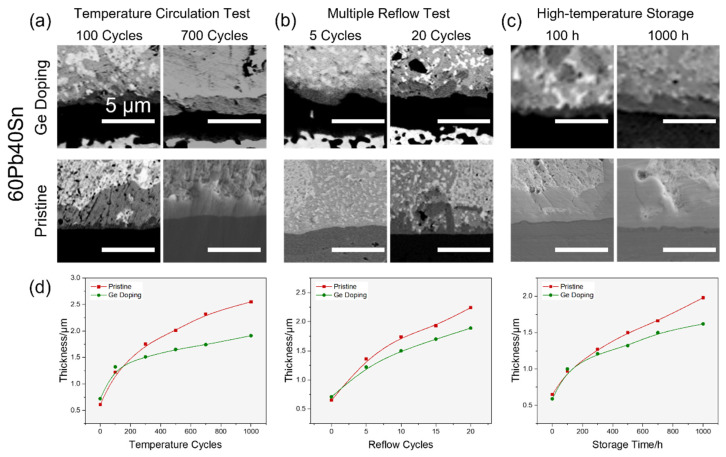
The reliability comparison between Pb59.97Sn40Ge0.03 solder with Ge doping and Pb60Sn40 without doping. The SEM images the IMC on the Ni-Cu substrate in the temperature circulation test (**a**), the multiple reflow test (**b**) and the high-temperature storage test (**c**). The IMC thickness evolutions in three reliability tests are displayed in (**d**).

To evidence the repression of Ge doping to the IMCs of PbSn solders, Pb59.97Sn40Ge0.03 solder (the concentration of Ge was 0.013 wt%) was fabricated using the same method shown in Figure S1a and compared with Pb60Sn40 solder without Ge doping in all three reliability tests, and the results are shown in Figure 7.

After 1000 temperature cycles, the Pb59.97Sn40Ge0.03 and Pb60Sn40 bumps maintained their interconnection structures and no obvious cracks were observed, but the IMC layers were much thicker during the test. The IMC layer was planar with Ge doping, while the IMC layer without Ge infiltrated into the solders fiercely when the temperature cycles exceeded 700. In the multiple reflow test, the initial planar IMC layers were both thin for the two samples, but the IMCs in the solder with Ge doping propagated more slowly. When the reflow cycles reached 20, the IMCs in the Pb59.97Sn40Ge0.03 solder showed a scallop-like shape while the IMCs in the Pb60Sn40 solder changed to a bamboo-shoot-like morphology, which was more brittle and easier to break under local stress. During high-temperature storage, the changes in IMC morphologies were relatively smaller. As the storage time increased from 100 h to 1000 h, the IMC layer maintained planar growth for Pb60Sn40. The IMC morphologies in Pb59.97Sn40Ge0.03 solder changed from coarse front to planar surface, exhibiting a roughness decrease. The spontaneous recovery from a rough interface to planar growth mode should be attributed to the growth kinetics and interface thermodynamics. At first, the IMC growth rate was higher, so the scallop-shaped IMC layer was more favorable. However, its ragged interface with the solder contained higher surface energy than the planar interface, driving the IMC–solder interface to transform into a planar shape. In the other two reliability tests where environment conditions changed continuously, similar phenomena were rarely observed.

According to the statistics of the IMCs thickness of the solders shown in Figure 7d, the IMC thickness in Ge-containing solders changed more slowly than Ge-less Pb60Sn40 solders, implying that the IMC growth was greatly suppressed by the trace Ge addition. The Ge-containing solders were less influenced by the failures caused by IMC growth, showing a better reliability and longer lifespan in all three reliability tests.

## 4. Conclusions

In this work, the microstructures, mechanical performances and reliability of pure PbSn solders and micro-alloyed PbSn solders with Ge were systematically investigated. The comprehensive observation of PbSn solders through metallographic analysis, tensile tests, wettability measurements and SEM images showed the performance evolution of PbSn solders with varying chemical compositions. In all three reliability tests of PbSn solders, it was verified that the Pb content contributed to the high reliability. Interfacial IMC (Ni_3_Sn_4_) growth and morphology evolution were responsible for reliability decrease. The Ge microalloying method was adopted to improve the reliability of PbSn solders. With 0.013 wt% Ge content instead of Pb in the Pb60Sn40 solder alloy, the IMC growth rate was largely suppressed, and the interfacial IMCs followed a planar growth instead of scallop-shaped or even bamboo-shoot-shaped fast growth mode, greatly reducing the risk of interconnect failure. First-principle calculation gave the mechanism of the IMC growth and explained the inhibition effect of Ge doping. The Ge was doped in the Ni_3_Sn_4_ lattice and partially intercepted the Ni-vacancies exchange diffusion by enhancing the exchange energy barrier. The IMC growth greatly relied on the exchange diffusion. Therefore, Ge microalloying efficiently elevated the reliability of PbSn solder by suppressing IMC growth. To sum up, the Ni_3_Sn_4_ IMC growth in PbSn solders can be greatly inhibited and delayed via the microalloying method using trace Ge owing to the decline in the atomic diffusion rate, which indicated that the Ge microalloying method can be effective for PbSn solders, contributing to the optimization of interconnects in products for aerospace and military applications. We believe that our research not only successfully fabricated highly reliable Ge micro-alloyed PbSn solders but also explained the underlying mechanisms during IMC evolutions. This study provides inspiration for optimizing the reliability of PbSn solders using microalloying methods.

## Figures and Tables

**Table 1:** The atomic coordinates of Ni and Sn atoms in the cell.

Coordinates	Ni (2a)	Ni (4i)	Sn (4i_1_)	Sn (4i_2_)
x	0	0.215	0.429	0.172
y	0	0	0	0
z	0	0.337	0.687	0.812

**Table 2:** The required energy for vacancy formation at different positions in the cell.

Vacancy Position	Ni (2a)	Ni (4i)	Sn (4i_1_)	Sn (4i_2_)
Vacancy Formation Energy (eV)	0.89870	0.84389	1.55615	1.45499

**Table 3:** The required energy for exchange between Ni atoms and the vacancies.

Diffusion Path	Exchange Energy Barrier/eV	Final Diffusion Path
1~0	0.43641	1~0
1~2	1.40215	
1~3	1.32652	
3~4	1.85745	
3~1	1.22524	

**Table 4:** The cell formation energy with/without doping.

Doping Position	Coordinates	Cell Formation Energy/eV	
		Ni_24_Sn_32_ (Ge)	Ni_24_Sn_32_ (Cu)
Ni (2a)	(0.5000, 0.2500, 0.5000)	−0.24012	−0.25769
Ni (4i)	(0.2850, 0.2500, 0.3315)	−0.23978	−0.26015
Sn (4i_1_)	(0.1720, 0.5000, 0.4060)	−0.25894	−0.25433
Sn (4i_2_)	(0.0710, 0.2500, 0.1565)	−0.25943	−0.25467
Without Doping		−0.26089	

**Table 5:** The required energy for Ni exchange after Ge doping.

Atomic Number	Diffusion Path	Exchange Energy Barrier/eV	Final Diffusion Path
a1	1~0	0.54846	1~0
	1~2	1.55834	
	1~3	1.44256	
a3	3~4	1.96845	
	3~1	1.36576	
a5 a1 a0 a2 a6	5~1	0.53698	a5~a1~a0~a2~a6
	1~0	0.54846	
	0~2	0.54720	
	2~6	0.53677	

**Table 6:** The required energy for Ni exchange after Cu doping.

Atomic Number	Diffusion Path	Exchange Energy Barrier/eV	Final Diffusion Path
a1	1~0	0.43024	1~0
	1~2	1.40024	
	1~3	1.29755	
a3	3~4	1.89857	
	3~1	1.28752	
a5 a1 a0 a2 a6	5~1	0.43231	a5~a1~a0~a2~a6
	1~0	0.43024	
	0~2	0.42684	
	2~6	0.43021	

## Data Availability

Data are contained within the article.

## Supplementary Materials

The following supporting information can be downloaded at: https://www.mdpi.com/article/10.3390/ma17030724/s1. Figure S1. The experiment method illustration. (a) The fabrication of solder alloys with the protection of melting salt. After the casting process, the alloy specimens were then analyzed by metallographic analysis and wettability measurement. (b) The applied shear stress detached the above flip chip from the substrate for observation of the bumps. (c) The circuit design of the package platform; Figure S2. The standard tensile sample used for the mechanical property measurement; Figure S3. The IMC evolution during temperature circulation test. The SEM images of the IMC on the Ni-Cu substrate of the three solder alloys: 90Pb10Sn, 60Pb40Sn, 37Pb63Sn; Figure S4. The IMC evolution during multiple reflow tests. The SEM images of the IMC on the Ni-Cu substrate of the three solder alloys: 90Pb10Sn, 60Pb40Sn, 37Pb63Sn; Figure S5. The IMC evolution during high-temperature storage. The SEM images of the IMC on the Ni-Cu substrate of the three solder alloys: 90Pb10Sn, 60Pb40Sn, 37Pb63Sn; Table S1. The first principles calculation parameters settings for vacancies formation energy; Table S2. The atomic radius of the elements; Table S3. The first principles calculation parameters settings for Ni-vacancies exchange energy barriers.
