# Supplementary material for: Improvement of PbSn Solder Reliability with Ge Microalloying-Induced Optimization of Intermetallic Compounds Growth
Source: Materials (Basel). 2024 Feb 2;17(3):724. doi: 10.3390/ma17030724 (PMC10856678; doi:10.3390/ma17030724)
Supplement: Supplementary file 1 [file materials-17-00724-s001.zip › materials-2746955-supplementary.pdf]

Article

Improvement of PbSn Solder Reliability with Ge Microalloying-Induced Optimization of Intermetallic Compounds Growth

Zhibo Qu [1,2], Yilong Hao [1], Changhao Chen [2], Yong Wang [2], Shimeng Xu [2,3], Shuyuan Shi [4], Pengrong Lin [2,5,*] and Xiaochen Xie [2,4,*]

1 School of Integrated Circuits, Peking University, Beijing 100871, China; quzhibo1991@hotmail.com (Z.Q.); haol@pku.edu.cn (Y.H.)
2 Package R&D Center, Beijing Microelectroniscs Technology Institute, Beijing 100076, China; chenchanghao0122@163.com (C.C.); wangy@mxtronics.com (Y.W.); xsm23@mails.tsinghua.edu.cn (S.X.)
3 School of Integrated Circuits, TsingHua University, Beijing 100084, China
4 Fert Beijing Institute, MIIT Key Laboratory of Spintronics, School of Integrated Circuit Science and Engineering, Beihang University, Beijing 100191, China; smeshis@buaa.edu.cn
5 Department of Mechanical Engineering, Tsinghua University, Beijing 100084, China
* Correspondence: lpr666@vip.163.com (P.L.); xiexiaochen@buaa.edu.cn (X.X.)

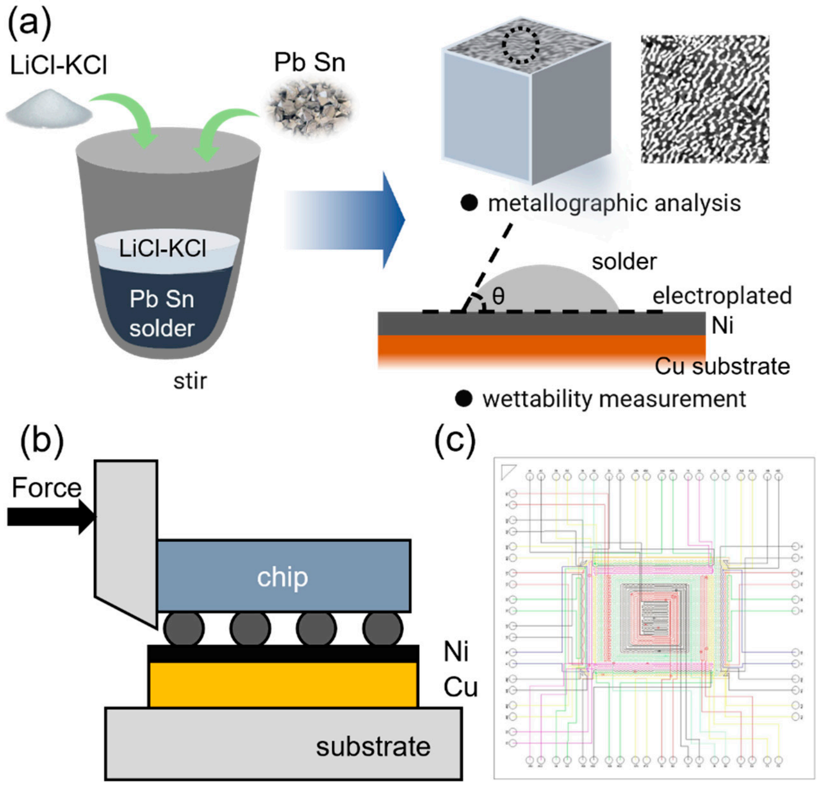

Figure S1. The experiment method illustration. (a) The fabrication of solder alloys with the protection of melting salt. After the casting process, the alloy specimens were then analyzed by metallographic analysis and wettability measurement. (b) The applied shear stress detached the above flip chip from the substrate for observation of the bumps. (c) The circuit design of the package platform.

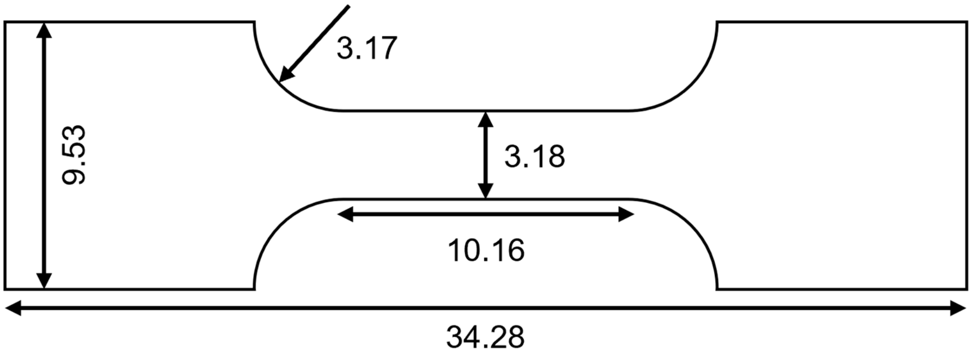

Figure S2. The standard tensile sample used for the mechanical property measurement.

The reliability test details:

Temperature Cycling Test:

The ETAC MLR22 conductivity reliability testing system was employed for high and low temperature cycling tests. Considering the melting point of the solder and national standard requirements, the high temperature section was set to 150 ℃ while the temperature section was set to -65 ℃. Each cycle consisted of two temperature sections and each section maintained for 10 minutes with a conversion time between the two temperature sections less than 20 s. The temperature cycling nodes were set as 0, 300, 700, 1000 times respectively.

Multiple Reflow Test:

The TYD KW600 reflux furnace produced by Beijing Keyadi Electronic Technology Co., Ltd. was used in this test. Before the first reflow, a uniform layer of OM-36 soldering flux should be applied to the surface of the solder; Subsequently, the sample was placed in the reflow furnace, with the three temperature zones set to 150 ℃, 220 ℃, and 220 ℃, respectively. The running speed of the reflux furnace track was 20-40 cm/min.

High-Temperature Storage Test:

The equipment used in this experiment was a drying oven produced by Shanghai Yiheng Scientific Instrument Co., Ltd. Referring to the American Solid State Technology Association standard JESD22-A103E "High Temperature Storage Life" and the melting point of the solder in this experiment, the experimental temperature was set to 125 ℃. The high-temperature storage time was set to 500 h and 1000 h.

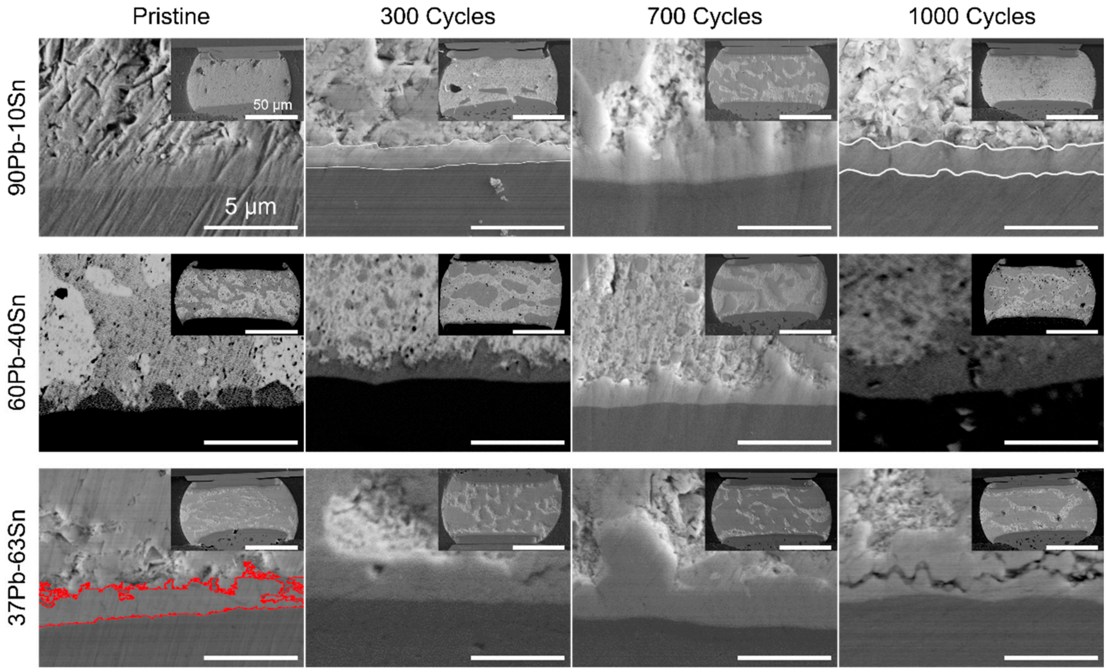

Figure S3. The IMC evolution during temperature circulation test. The SEM images of the IMC on the Ni-Cu substrate of the three solder alloys: 90Pb10Sn, 60Pb40Sn, 37Pb63Sn.

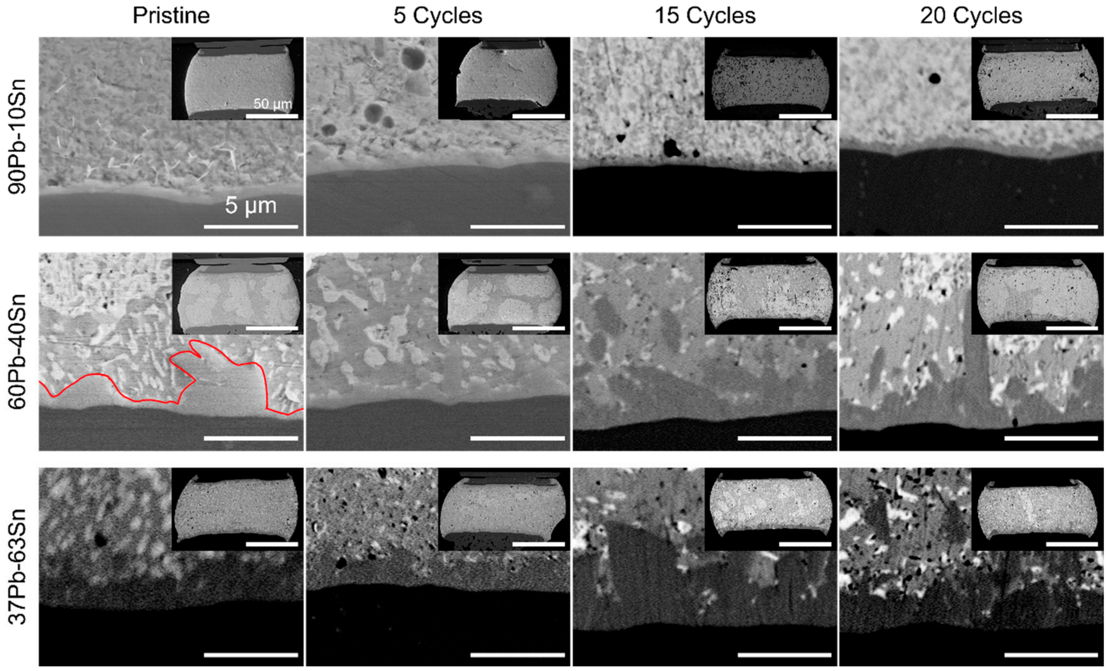

Figure S4. The IMC evolution during multiple reflow tests. The SEM images of the IMC on the Ni-Cu substrate of the three solder alloys: 90Pb10Sn, 60Pb40Sn, 37Pb63Sn.

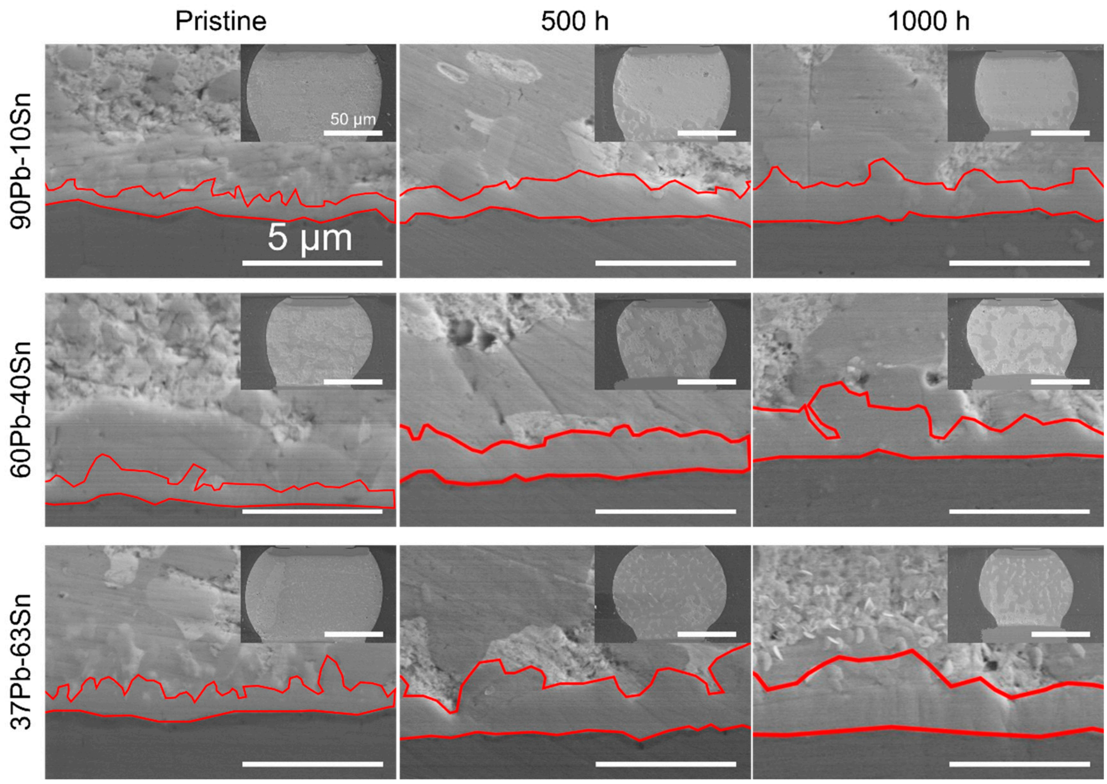

Figure S5. The IMC evolution during high-temperature storage. The SEM images of the IMC on the Ni-Cu substrate of the three solder alloys: 90Pb10Sn, 60Pb40Sn, 37Pb63Sn.

Table S1. The first principles calculation parameters settings for vacancies formation energy.

Calculation accuracy	k-point	Energy Cut-off	convergence break condition	
Fine	2×6×5	300 eV	1×10^{-5} eV/atom (Energy)	0.03 eV/Å (stress)

Table S2. The atomic radius of the elements.

Atom	Atomic number	Measured atomic radius (Å)
Sn	20	1.58
Pb	82	1.75
Ni	28	1.24
Cu	29	1.28
Ge	32	1.40

Table S3. The first principles calculation parameters settings for Ni-vacancies exchange energy barriers.

Supercell	k-point	Energy Cut-off	convergence break condition	
1×2×2	2×5×4	270 eV	2×10^{-5} eV/atom (Energy)	0.05 eV/Å (stress)
